# Similar negative effects of fatigue on physical activity in persons with rheumatoid arthritis and healthy controls: results of a cross-sectional patient-control study

**DOI:** 10.1016/j.ero.2026.100185

**Published:** 2026-05-07

**Authors:** Maartje Cox, Sofia Ramiro, Kyra Theunissen, Kenneth Meijer, Annick Timmermans, Annelies Boonen, Guy Plasqui

**Affiliations:** 1 Department of Nutrition and Movement Sciences, School of Nutrition and Translational Research in Metabolism, Maastricht University, Maastricht, The Netherlands; 2 Australian National Phenome Centre, Health Futures Institute, Harry Perkins Institute of Medical Research, Murdoch University, Perth, Western Australia, Australia; 3 Department of Rheumatology, Leiden University Medical Center, Leiden, The Netherlands; 4 Department of Rheumatology, Zuyderland Medical Centre Heerlen, Heerlen, The Netherlands; 5 Department of Internal Medicine, Division of Rheumatology, Maastricht University Medical Center, Maastricht, The Netherlands; 6 Department of Health Services Research, Care & Public Health Research Institute (CAPHRI), Maastricht University, Maastricht, The Netherlands; 7 REVAL Rehabilitation Research Center, REVAL, Faculty of Rehabilitation Sciences, Hasselt University, Hasselt, Belgium

## Abstract

**Objectives:**

The objective of this study was to investigate objectively measured physical activity (PA) patterns and the association between PA and self-reported fatigue in persons with rheumatoid arthritis (pwRA), as compared with healthy controls (HCs).

**Methods:**

In this cross-sectional study, 23 pwRA (Disease Activity Score 28 > 2.6, 52 ± 13 years, 20 females) and 18 HCs (52 ± 10 years, 14 females) wore an ActiGraph wGT3X-BT tri-axial accelerometer (ActiGraph LLC) on 1 ankle for at least 4 days. Accelerometer-based outcomes were vector magnitude counts/min (VM_CPM_), step count, and the amount of time that PA was performed at moderate-to-vigorous intensity (MVPA). Accelerometer data were categorised by day (weekday vs weekend) and time of the day (daytime vs evening) to investigate PA patterns. Physical and cognitive fatigue were assessed with questionnaires (Fatigue Severity Scale and Modified Fatigue Impact Scale). Associations between fatigue, presence of RA, and PA were examined using multilevel generalised estimating equations models, with patient and day-timing as levels. Interactions between the presence of RA and fatigue were tested.

**Results:**

The VM_CPM_, step count, and MVPA were lower in pwRA than in HC across various daytime periods (overall: 1233.4 ± 509.4 cpm/h in pwRA vs 1701.2 ± 492.8 in HC, 586.9 ± 225.8 vs 723.8 ± 224.1 steps/h, and 1.6 ± 1.4 vs 2.7 ± 1.5 min/h, respectively). Multivariably, lower VM_CPM_ was seen in pwRA (β = −330.63; 95% CI = −650.45 to −10.81), with higher fatigue (β = −43.89; 95% CI = −135.26 to 47.48), and with higher body mass index (β = −27.18; 95% C I = −50.73 to −3.62). The effect of fatigue or daytime on PA was not modified by the presence of RA.

**Conclusions:**

Persons with RA performed less PA than HC. Fatigue had a similarly negative effect on PA in pwRA and HC.

**WHAT IS ALREADY KNOWN ON THIS TOPIC**

• Physical activity (PA) can have positive effects on physical outcomes in people with rheumatoid arthritis (pwRA); however, patients are less active than their healthy counterparts. Fatigue—a symptom commonly reported by pwRA—may play a role in this, but relationships between fatigue and objectively quantified PA in pwRA remain largely unknown.

**WHAT THIS STUDY ADDS**

• We found that physical fatigue negatively affects PA in pwRA and healthy controls (HCs), with no difference in the extent to which physical fatigue influences PA between the 2 groups. Objectively quantified PA was lower in pwRA than in HC, regardless of the day of the week (week/weekend) or the time of day (day/evening). Differences between patients and controls were smallest on weekdays during daytime, and largest during weekday evenings and on weekend days during daytime. Body mass index (BMI) was negatively associated with PA outcomes.

**HOW THIS STUDY MIGHT AFFECT RESEARCH, PRACTICE OR POLICY**

• Future research should investigate patients’ self-reported levels of fatigue longitudinally to elucidate potential causal relationships between fatigue and PA in pwRA. BMI management in pwRA appears relevant for clinical practice.

Alt-text: Unlabelled box dummy alt text

## INTRODUCTION

Fatigue is a common, yet complex and incompletely understood, symptom in rheumatoid arthritis (RA) [1], and previous research has highlighted the positive effects of physical activity (PA) on pain, physical functionality, and muscle function without inducing adverse effects on disease activity [2,3]. Importantly, PA is part of the nonpharmacologic management of RA [[4], [5], [6]]. However, persons with RA (pwRA) may feel demotivated to engage in PA due to perceived fatigue and are therefore prone to fall short of the World Health Organization’s PA recommendations [7,8].

Accurate quantification of the amount of daily PA is vital but challenging, as subjectively reported PA using questionnaires has poor reliability and validity [9]. Self-reported high-intensity PA and sedentary time were higher and lower, respectively, in pwRA than when measured objectively [10], thus indicating a need for objective measurement of PA. Accelerometry offers an objective and validated method for the quantification of PA in free-living conditions [9]. Moreover, it provides an opportunity to categorise the registered PA into different intensities by applying a set of threshold values, or ‘cut-points’ [5,9]. There is a growing number of studies utilising accelerometry to investigate PA in daily life in pwRA. Amongst others, these studies found significantly more sedentary behaviour [6] and significantly less PA performed at moderate-to-vigorous intensity (MVPA) [11,12] in pwRA than in healthy controls (HCs). Most PA in pwRA is detected at light and very light intensities (ie, 2.0-2.9 and 1.1-1.9 metabolic equivalents, respectively) [13]. Even at such low intensities, PA is inversely related to 10-year cardiovascular disease risk, limitations in physical functionality, and RA disease activity [13,14]. Additionally, past research found lower physical fatigue in pwRA who self-reported to be regularly physically active (ie, who performed moderate- to high-intensity PA for ≥3 hour per week or high-intensity exercise for ≥4 hour per week) than in pwRA who self-reported to be mainly sedentary or engaging in irregular low-to-moderate-intensity exercise [15].

The relationship between self-reported fatigue and objectively quantified PA through accelerometry in pwRA, as compared with HC, remains largely unknown, as is the PA pattern of pwRA throughout the day and during the week. Furthermore, fatigue is a complex symptom with an often-unknown aetiology, which can be separated into physical and cognitive domains. With >70% of pwRA experiencing excessive fatigue [16], it is of importance to further explore associations between fatigue and PA, while taking the timing of PA into account [17]. Understanding this association will offer a starting point for future research investigating causality and interventions to improve fatigue. Therefore, the present study aimed to investigate the association between self-reported fatigue and objectively measured PA, taking PA patterns into account, in pwRA as compared with HC.

## METHODS

The present cross-sectional observational study was conducted as part of a larger research project investigating PA, gait parameters, cost of walking, and fatigue in pwRA compared with HC [18]. Ethical approval was obtained from the Medical Ethics Committee of Maastricht University Medical Center+ (MUMC+, the Netherlands) (NL72955.068.20), and the study procedure was compliant with the Declaration of Helsinki. Written informed consent was obtained from all participants.

### Participants

Participants were eligible for inclusion in the RA group if they were diagnosed with RA by a rheumatologist, between 18 and 70 years of age, and had active disease or consequences thereof (Disease Activity Score 28 > 2.6 or erosions in the feet) [19]. PwRA were recruited from the MUMC+ and Zuyderland Hospital (Sittard-Geleen, Netherlands) outpatient clinics. Age-, sex-, and body mass index (BMI)-matched HC were recruited via information flyers in the MUMC+. Exclusion criteria for both groups were arthroplasty of the hip, knee, ankle or shoulder, severe osteoarthritis of the hip, knee, ankle or shoulder with an indication for surgery, fracture in the lower limbs affecting gait, contraindication for PA as assessed by a medical doctor, comorbidities affecting gait or metabolism (eg, chronic obstructive pulmonary disease or Parkinson disease), a dependency on walking aid or orthosis for walking, and fixation of the lumbar or cervical spine. All participants were recruited, and data were collected in the period between April 2021 and July 2022.

The sample size for this research project was determined based on the original research aim, which was to evaluate the cost of walking in pwRA and HC. A 2-tailed *a priori* independent t-test was conducted to compare the Metabolic Equivalent Rates of pwRA and control subjects at 4 km/h. Statistical power was set at 80% (1−β = .80), with a significance level of 5% (α = .05). Allowing for an anticipated dropout rate of 5%, the required total sample size was estimated at 30 pwRA and 30 control subjects. A total of 50 participants (28 pwRA and 22 HCs) were included in the main study of the current research project, but only 41 consented to participating in the accelerometry-based substudy that is currently presented.

### Accelerometry

PA in daily life was assessed by accelerometry using an ActiGraph wGT3X-BT tri-axial accelerometer (ActiGraph LLC). The ActiGraph is one of the most widely validated accelerometers and has repeatedly been shown to be a reliable device for the assessment of daily PA [[20], [21], [22], [23]]. Participants were instructed to wear the ActiGraph on the ankle of the preferred side (left/right) for 7 days during waking hours and to remove the device when showering, swimming, or sleeping. Data were collected at a sampling frequency of 30 Hz. Accelerometer data were downloaded and aggregated into 60-second epochs (ActiLife version 6.13.4, ActiGraph LLC). Nonwear periods were identified with the Choi algorithm [24], as this algorithm was previously recommended for usage on cohorts of older adults [9]. Briefly, the Choi algorithm defines nonwear as 90 minutes of consecutive zero counts, with a spike tolerance of 2 minutes (ie, 2 minutes of artifactual nonzero counts are allowed), and a 30-minute small window length (indicating that the 30 minutes before and after an artifactual movement interval must show consecutive zero counts) [24]. This definition is in line with the recommendation of Semanik et al [5] to allow nonmovement time periods of 90 minutes in RA populations before classifying accelerometer data as nonwear.

Inspection of the accelerometer data revealed that some participants wore the device overnight and for less than 7 days, despite instructions. Only accelerometer data collected between 6:30 AM and 11:59 PM were considered for analysis to eliminate any influence of PA registered during the night. Minimum daily wear time and minimum number of valid wear days per participant were set to 600 minutes and 4 days, respectively [5,9].

Accelerometer outcomes were vector magnitude counts per minute (VM_CPM_ in mean counts/min per hour), the time (in minutes per hour) that participants performed PA at moderate-to-vigorous intensity (MVPA), and step count (ie, steps per hour). The accelerometer registered movement on 3 axes: vertical (X), frontal (Y), and sagittal (Z). VM_CPM_ was calculated as the square root of the sum of all 3 axes’ cpm squared (Equation 1) using ActiLife software (ActiGraph LLC). Step count was automatically determined by a preprogrammed algorithm in the ActiLife software. To determine the time spent exercising at MVPA, the cut-points for ankle-worn accelerometers as proposed by Bammann et al were employed [25], with different cut-points for men and women. The mean values of the proposed cut-points for the dominant and nondominant ankle were used as dominance was not determined in the present population [25].

(1) $$\text{Vector}\;\text{magnitude}\;\text{cpm}\;=\;\sqrt{X-\text{axis}\;\text{cp}m^{2}+\;Y-\text{axis}\;\text{cp}m^{2}+\;Z-\text{axis}\;\text{cp}m^{2}}$$

Previous research has shown that a minimum of 3 days is required to obtain accelerometer data able to reliably predict PA at light, moderate, and vigorous intensities in older adults [26,27]. Participants of this study wore the accelerometer on at least 4 days, for at least 10 hours per day, thus ensuring that our results represent a typical PA pattern. Furthermore, PA patterns are often different on weekdays than on weekend days. We captured these differences by dividing the collected data into weekdays (ie, Monday to Friday) and weekend days (ie, Saturday and Sunday) as well as daytime (6:30 AM until 5:00 PM) and evening (5:01 PM until 11:59 PM). Three dummy variables were created to indicate the timing of PA registration: (i) day (with weekend = 1 and week = 0), (ii) timing (with evening = 1 and daytime = 0), and (iii) day-timing (with 4 levels: weekend-daytime (reference), weekday-daytime, weekend-evening, and weekday-evening).

### Clinical assessments

Sex, age, and BMI were collected from all participants. The number of self-reported painful joints was assessed using a body diagram. In total, 42 joints were assessed (21 on each side), comprising the shoulder, elbow, wrist, hip, knee, ankle, metacarpophalangeal joints, proximal interphalangeal joints, and toe joints.

All participants completed the Fatigue Severity Scale (FSS) and the Modified Fatigue Impact Scale (MFIS) [28]. The FSS is focused on fatigue affecting daily life functioning in the physical domain and consists of 9 items scaled from 1 to 7. The MFIS includes 21 items on the extent to which fatigue impacts daily life in physical, cognitive, and psychosocial domains, with items being scored from 0 to 4 [29,30]. Results from these questionnaires were employed to investigate the influence of fatigue on PA in pwRA and HC. Participants also completed the Health Assessment Questionnaire-Disability Index (HAQ-DI) regarding their functional status, with scores ranging from 0 to 3 and higher scores indicating more functional impairment [31].

### Statistical analyses

Participant characteristics and accelerometry outcomes were compared between pwRA and HC using Mann-Whitney U tests. To investigate the role of fatigue and day-timing of activities on the association between group (pwRA vs HC) and PA, generalised estimating equations (GEEs) were employed, taking potential confounders into account. Models were computed for the 3 accelerometer outcomes (ie, VM_CPM_, MVPA, and step count) separately. Univariable and multivariable GEE models utilising the ‘exchangeable’ correlation structure were created, with participant and day-timing as levels in the analyses. First, a univariable model was constructed with group (pwRA vs HC) as the predictor to determine its coefficient univariably. Subsequently, multivariable models were created with age, sex, BMI, FSS, MFIS physical domain, MFIS cognitive domain, day (ie, week or weekend), timing (ie, daytime or evening), day-timing (ie, week-daytime, week-evening, weekend-daytime, weekend-evening) as potential explanatory variables. Variables were included in the final GEE model if they significantly predicted the outcome or if they altered the coefficient for the group with >10%. If fatigue measured with the FSS and with the MFIS questionnaires were both confounders or explaining the outcome, then separate final GEE models were created (ie, 1 with FSS and 1 with MFIS) as these variables measure the same construct and should thus not be included in the same model. Finally, interactions between group and individual predictor variables were tested for all variables assessed, and the analysis was stratified if such interactions were statistically significant and clinically relevant. Multiplicity correction was not applied. The classification of timing (ie, by day and by time of day) into 4 categories was handled as a dummy variable, thus reducing the risk for type 1 errors. Statistical analyses were performed in R (version 4.3.3, R Foundation for Statistical Computing), and the level for statistical significance was set to *P* ≤ .05.

## RESULTS

Out of the 28 pwRA and 22 HCs that took part in the main study of this research project investigating gait and the cost of walking, 23 pwRA and 18 age-, sex-, and BMI-matched HC also consented to participate in the present study (Table 1). Self-reported functional status (HAQ-DI) and fatigue (FSS and MFIS) differed significantly between pwRA and HC, with higher levels of disability and fatigue reported by patients. Following the match at inclusion, age, sex, and BMI did not differ between groups. Four HCs did not complete the FSS, MFIS, and HAQ-DI questionnaires, while 1 pwRA did not fill out the HAQ-DI. The GEE models used in this study are generally considered sufficiently robust to handle a minimal number of missing values; therefore, missing data were not imputed.

**Table 1:** Participant characteristics

Group	pwRA (n = 23)	HC (n = 18)
Female	20 (87%)	14 (78%)
Age (y)	52 (13) [24-68]	52 (10) [23-64]
Weight (kg)	74.1 (13.2) [55.6-113.1]	71.4 (13.1) [49.7-95.2]
Height (cm)	168.1 (6.3) [157.0-181.5]	170.8 (8.8) [155.0-190.0]
BMI (kg/m^2^)	26.2 (4.4) [21.3-42.2]	24.4 (3.4) [18.9-29.7]
HAQ-DI (0-3)^a^	0.9 (0.7) [0-2.3]	0.0 (0.2) [0-0.6]^b^
Number of painful joints (0-42)	8.3 (10.9) [0-42]	c
FSS (0-7)^a^	4.7 (1.4) [1-7.0]	2.1 (1.0) [1-3.7]^b^
MFIS physical (0-36)^a^	17.0 (8.7) [0-31]	2.5 (4.7) [0-13]^b^
MFIS cognitive (0-40)^a^	16.2 (8.4) [2-35]	7.0 (8.8) [0-23]^b^

BMI, Body Mass Index; FSS, Fatigue Severity Scale; HAQ-DI, Health Assessment Questionnaire-Disability Index; HC, healthy control; MFIS, Modified Fatigue Impact Scale; pwRA, persons with rheumatoid arthritis.

Data displayed as mean (SD) [range] or n (%).

a Four HCs did not complete the FSS, MFIS, or HAQ-DI questionnaires. One pwRA did not fill out the HAQ-DI.

b *P* < .05 between HC and pwRA.

c Painful joints were not evaluated in HC.

### Accelerometry outcomes

Despite instructions to take the device off before going to sleep, data showed sporadic nightly wear time with or without activity for several participants. In these instances, the applied time filter removed activity registered during the night. Four participants had worn the accelerometer for less than 600 minutes on a particular day, and these days were therefore excluded from further analyses. As all participants met the minimum wear time requirements of at least 4 days of 600 minutes each, no participants were excluded from further analysis. Overall, 263 days of accelerometer wear were analysed, of which 55% regarded data from the RA group. All participants except 2 (1 from each group) wore the device on 1 or more weekend day(s), yielding a total of 74 weekend days (57% from the RA group) and a total of 189 weekdays (54% from the RA group).

**Figure 1:**
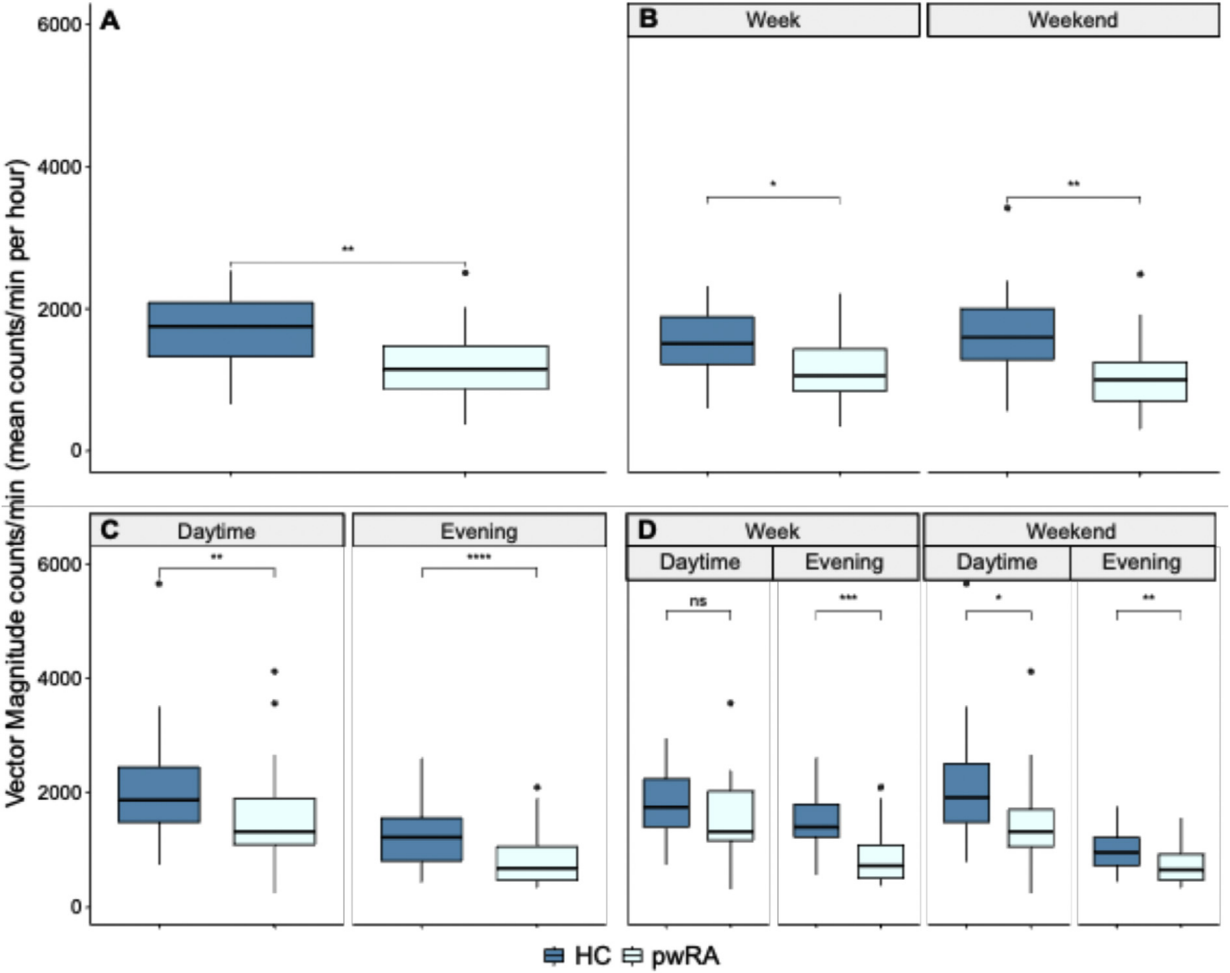
Vector magnitude counts per minute (mean counts/min per hour) measured in persons 1 (A), on weekdays as compared with the weekend (B), during the day and during the evening (C), and separated by day as well as timing of the day (D). ns, *P* > .05, *P < .05, **P < .01, ***P < .001, ****P < .0001. Specific values are provided in Supplementary Tables S1 to S3. HC, healthy control; pwRA, persons with rheumatoid arthritis.

**Figure 2:**
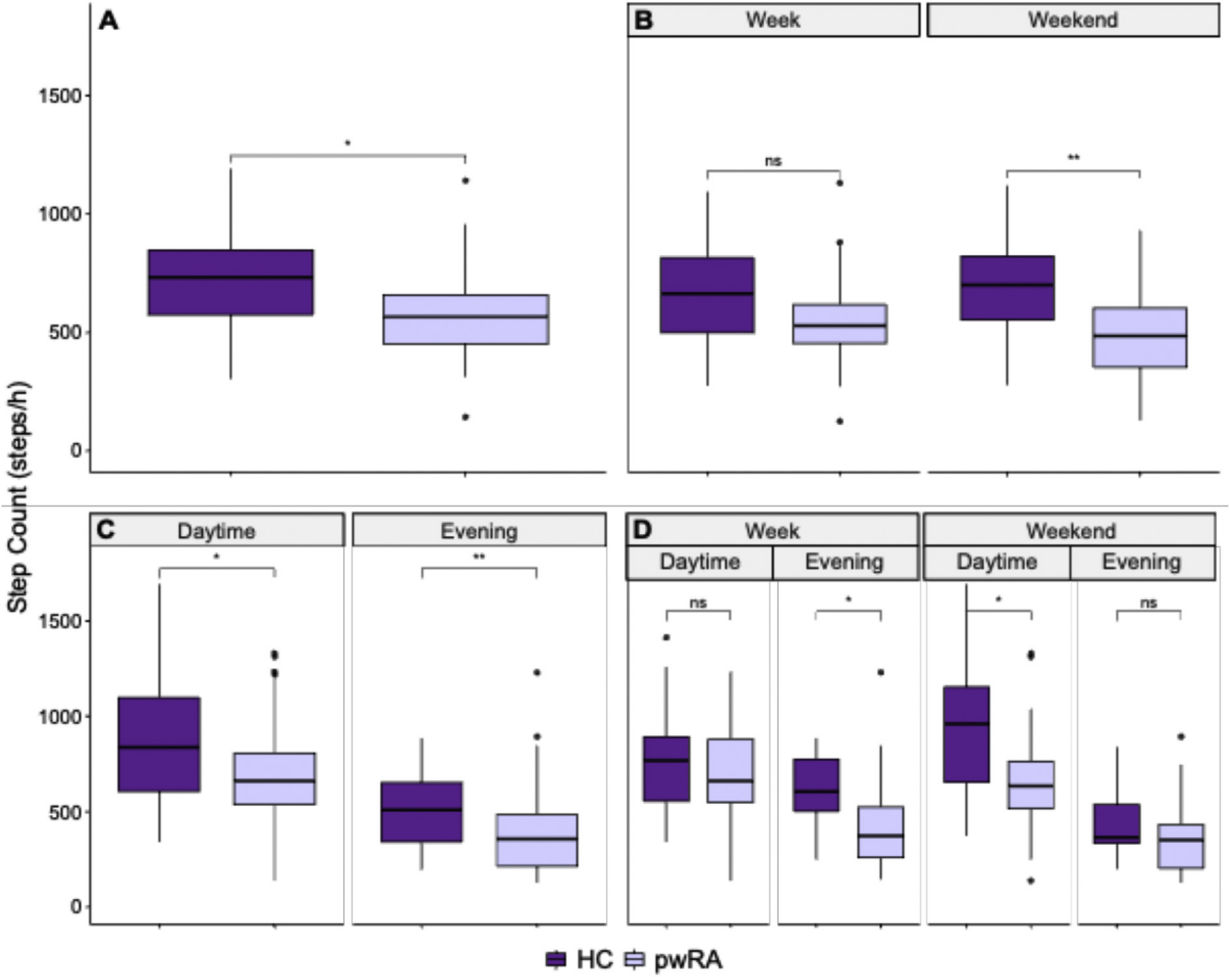
Number of steps taken per hour by persons with rheumatoid arthritis (pwRA) and healthy controls (HCs) in general (A), on weekdays vs on weekend days (B), during the day as compared with the evening (C), and separately on weekdays or weekend days as well as timing of the day (D). ns, *P* > .05, *P < .05, **P < .01. Specific values are provided in Supplementary Table S1 to S3.

The VM_CPM_ was significantly lower in pwRA than in HC (*P* = .004, Fig 1A and Supplementary Table S1), indicating reduced PA in pwRA. This observation persisted after stratifying the data according to the day of collection (ie, on weekdays *P* = .02 and on weekend days *P* = .004, Fig 1B and Supplementary Table S1) and according to the time of collection (ie, during the day *P* = .003 and during the evening *P* < .001, Fig 1C and Supplementary Table S2). When stratifying the data according to day and time simultaneously, VM_CPM_ did not differ between pwRA and HC during the day on weekdays (*P* = .12, Fig 1D and Supplementary Table S3). In line with the pattern of VM_CPM_, the number of steps per hour was consistently lower for pwRA than for HC (Fig 2). The numeric difference in VM_CPM_ and step count between pwRA and HC was largest on week evenings (ie, 880.7 [491.4] in pwRA vs 1457.3 [572.7] in HC, *P* < .001 for VM_CPM_ and 427.5 [253.9] in pwRA vs 594.0 (197.6) in HC, *P* = .01 for step count) and on weekends during daytime (ie, 1453.1 [819.7] in pwRA vs 2227.7 [1162.9] in HC, *P* = .01 for VM_CPM_ and 672.5 [295.4] in pwRA vs 923.2 [346.7] in HC, *P* = .03 for step count, Supplementary Table S3).

**Figure 3:**
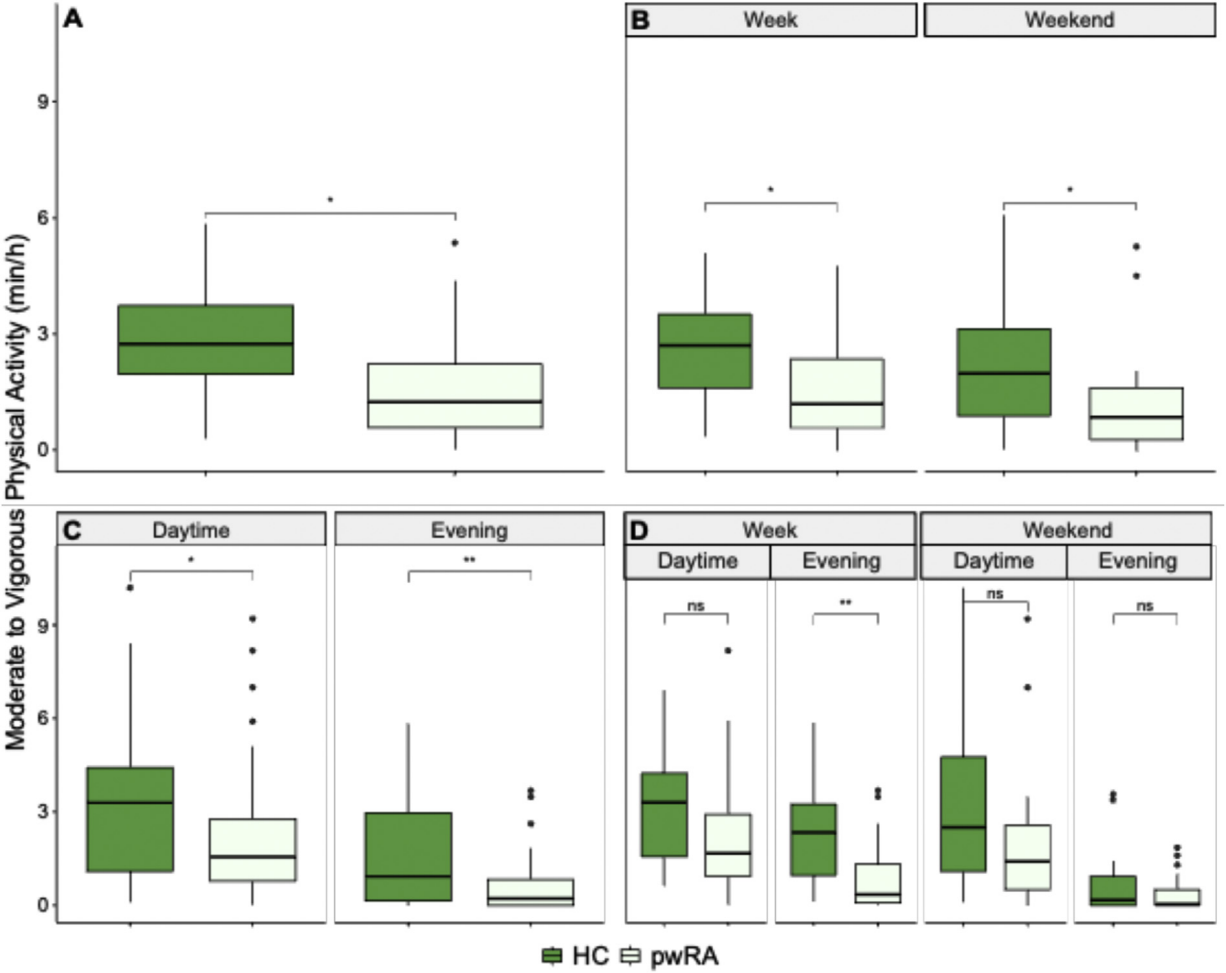
Minutes per hour with physical activity performed at moderate-to-vigorous intensity by persons with rheumatoid arthritis (pwRA) and healthy controls (HCs) overall (A), on weekdays as compared to the weekend (B), during the day and during the evening (C), and separated by day as well as timing of the day (D). ns, *P* > .05, *P < .05, **P < .01. Specific values are provided in Supplementary Tables S1 to S3.

Finally, MVPA was also reduced in pwRA compared with HC (*P* = .02, Fig 3). Differences between the 2 groups were largest on week evenings (0.9 [1.1] in pwRA vs 2.4 [1.6] in HC, *P* = .001) and smallest on weekend evenings (0.4 [0.6] in pwRA vs 0.7 [1.1], *P* = .4, Supplementary Table S3). Values for VM_CPM_, step count, and MVPA corresponding to Figure 1, Figure 2, Figure 3 are provided in Supplementary Tables S1 to S3.

### Modelling the relationship between fatigue, RA, and PA

#### VM_CPM_

The final multivariable GEE model included FSS (confounder of group), as well as BMI and day-timing (significantly associated with VM_CPM_ even after correction for group) in addition to the grouping variable (pwRA vs HC). No interactions were found between group and either of the remaining explanatory variables. The multivariable GEE model revealed that being diagnosed with RA, higher levels of fatigue severity, and higher BMI were negatively associated with VM_CPM_ (Table 2). More specifically, VM_CPM_ decreased by 330.63 cpm per hour in the presence of RA diagnosis, by 43.89 per point increase on the FSS scale, and by 27.18 cpm per unit increase in BMI. Moreover, compared with VM_CPM_ on weekend days (reference), PA was lower (numerically in stronger order) on weekdays during the day, weekdays during the evening, and weekend days during the evening.

**Table 2:** Effect of RA vs HC on the vector magnitude counts per minute (VM_CPM_) in univariable and multivariable generalised estimating equations analyses

	Univariable model	Multivariable model with FSS	Multivariable model with MFIS physical
Age (y)	2.48 (−9.53 to 14.50)	na	na
Male vs female	136.46 (−315.00 to 587.91)	na	na
BMI (kg/m^2^)	−47.14 (−78.60 to −15.67)	−27.18 (−50.73 to −3.62)	−28.69 (−52.22 to −5.15)
RA vs HC	−467.72 (−768.40 to −167.05)	−330.63 (−650.45 to −10.81)	−383.27 (−708.31 to −58.24)
FSS (0-7)	−117.06 (−216.04 to −18.09)	−43.89 (−135.26 to 47.48)	–
MFIS physical (0-36)	−17.23 (−33.50 to −0.96)	–	−4.04 (−19.56 to 11.48)
MFIS cognitive (0-40)	−13.85 (−27.83 to 0.12)	Na	na
Day (weekend vs week)	−24.48 (−301.03 to 252.07)	a	a
Timing (evening vs daytime)	−724.93 (−945.42 to −504.44)	a	a
Day-timing:			
Weekend-daytime	Reference	Reference	Reference
Weekday-daytime	−123.89 (−509.31 to 261.54)	−129.66 (−527.19 to 267.87)	−129.66 (−526.98 to 267.66)
Weekday-evening	−687.07 (−1059.60 to −314.54)	−723.68 (−1091.19 to −356.16)	−723.68 (−1092.34 to −355.02)
Weekend-evening	−942.19 (−1287.31 to −597.07)	−970.77 (−1322.72 to −618.82)	−970.77 (−1323.33 to −618.21)

Data presented as Coefficient (95% Confidence Interval).

BMI, Body Mass Index; FSS, Fatigue Severity Scale; HC, healthy control; MFIS, Modified Fatigue Impact Scale; RA, HC, healthy control; VMcpm, vector magnitude counts per minute.

na: variable not significant in the univariable model and not a confounder for the relationship between RA vs HC and Vector Magnitude counts per minute, and therefore not included in the multivariable model.

a Variables Day (Weekend vs Week) and timing (Evening vs Daytime) were not considered for the multivariable model, since the variable day-timing combines the information from both these variables.

The model with the physical domain of the MFIS instead of the FSS showed overall similar results, except that the negative impact of MFIS physical on VM_CPM_ was not significant.

#### Step count

The multivariable GEE model for step count included BMI, FSS, MFIS physical, Day, and timing (confounders for group) in addition to the grouping variable (pwRA vs HC). BMI, timing, and day-timing were significant predictors for step count after adjusting for group. Because day-timing combines the information provided by the Day and timing variables, the latter 2 were not included in the multivariable model. There were no interactions among the predictor variables.

RA diagnosis, BMI, and fatigue severity measured with the FSS were inversely associated with step count (coefficients of 0.46, −13.98, and –43.59, respectively; Supplementary Table S4). Furthermore, the number of steps taken per hour decreased as compared with weekend days during daytime in the following order: weekdays during the day (−6.03), weekdays in the evening (−302.06), and weekend days in the evening (−381.98) (Supplementary Table S4). A second multivariable model, with the physical domain of the MFIS questionnaire instead of FSS, revealed similar results, with negative effects on step count of RA diagnosis, higher BMI, and more severe physical fatigue, and a reduced number of steps on weekday-daytime, weekday-evening, and weekend-evening as compared with weekend-daytime (Supplementary Table S4).

#### Time in moderate-to-vigorous PA

In the multivariable model for MVPA, FSS and the physical domain of the MFIS (confounders for group), as well as BMI, and day-timing (significantly associated, after adjustment for group), were included. Timing was also significantly associated with MVPA after adjustment for group; however, information from this variable is covered by the day-timing variable. Age interacted significantly with group, and multivariable models were therefore stratified by median age (55 years), revealing a larger negative impact of RA diagnosis on MVPA in younger than in older patients (Supplementary Table S5).

Similar to what was found in the models for VM_CPM_ and step count, BMI, RA diagnosis, and fatigue severity (FSS and MFIS physical) were inversely associated with MVPA in older participants. In younger participants, BMI and fatigue were positively associated, but RA diagnosis was negatively correlated with MVPA. Across both age groups, MVPA was lower on weekdays and weekend days in the evening as compared with weekend days during daytime (Supplementary Table S5).

## DISCUSSION

We investigated objectively measured PA and its relationship with self-reported fatigue in pwRA versus HC. PA levels were lower in pwRA than HC across all days of the week and at any time of the day. Absolute differences in PA outcomes were smallest on weekdays during the day and largest during weekday evenings and on the weekend during daytime; however, when exploring relative differences, multilevel GEE models did not reveal significant group-by-time interactions, thus not confirming more exacerbated differences between groups on week evenings and on weekends during daytime. Fatigue showed a negative association with PA, but there was no significant interaction between fatigue and group, indicating that this effect was not stronger in pwRA as compared to HC.

Regarding the PA levels on different days and at different times of the day, we found significantly higher levels of PA in HC than in pwRA during the day (although not on weekdays), in line with previous research [6]. As opposed to what was reported before, HC in this study also performed more PA than pwRA in the evenings [6]. Variation in the considered time windows might underlie differences in the findings, as ‘evening’ was defined as the period from 6 PM to 9 PM in a previous study but as 5:01 PM to 11:59 PM in this study [6].

Absolute differences were smallest on weekdays during the day (VM_CPM_, step count, and MVPA were 17%, 8%, and 31% higher in HC than pwRA, respectively) and largest during weekday evenings (65%, 39%, and 170% higher in HC than pwRA, respectively) and on weekend days during daytime (53%, 37%, and 76% higher in HC than pwRA, respectively). A potential explanation for the differences in PA patterns might be found in similarities and differences in the constraints of day-to-day chores, resulting in similar PA levels for work and household tasks during the day from Monday to Friday. In the evenings and on the weekends, RA participants might experience higher levels of fatigue than HC participants from the demands of the past 5 days and therefore require time to recover, resulting in less PA during evening hours on weekdays and during daytime on Saturday and Sunday. Results of the present study cannot confirm nor reject this hypothesis as fatigue was only assessed cross-sectionally, and it was not determined whether participants indeed worked from Monday to Friday with Saturday and Sunday off. Unfortunately, data on the employment of our participants contained too many missing values to form meaningful knowledge.

We also investigated PA parameters, step count, and MVPA. Step count was found to be somewhat higher in the present study as compared with previous research (ie, on average 9298 vs 7452 steps/day in pwRA in present and past research, respectively [12]). However, HC participants of the present study also yielded a higher step count than those in past research (ie, 10,446 steps/d versus 11,323 steps/d), indicating that there may be a population bias between the British and Dutch populations under investigation, respectively. Total daily MVPA has been reported to be lower in pwRA than in HC [11,32], which our findings confirmed.

We found inverse associations between fatigue (particularly physical fatigue) and PA; however, as there were no interactions between the presence of RA and physical or cognitive fatigue in the multilevel GEE models, we did not find evidence that fatigue has a larger negative influence on PA in pwRA than in HC. Nonetheless, absolute fatigue levels were significantly higher in the RA than in the HC population, with inherently lower absolute associated PA levels, indicating a possible larger role played by fatigue in pwRA than in HC. Although the results of the present study do not allow us to draw any causal conclusions regarding the relationship between fatigue and PA (ie, whether pwRA perform less PA due to fatigue [mediation] or whether engaging in PA leads to increased fatigue), we hypothesize that the speed at which PA is performed may contribute to fatigue in pwRA. In laboratory-based gait analyses of the present cohort, we found that patients’ self-selected (preferred) walking speed is lower than their optimal walking speed (ie, the speed at which the walking-induced energy expenditure is lowest), while in HC, the preferred and optimal walking speeds were the same [18]. This implies that walking at the preferred walking speed during the day is associated with a higher energetic cost in pwRA, which in turn may promote feelings of fatigue. Interestingly, only the physical, but not the cognitive, domain of the MFIS fatigue questionnaire was a confounder for the relationship between group (RA vs HC) and PA in the multivariable models for step count and MVPA, indicating a differential role for fatigue subtypes in the effect on PA.

Our multivariable models did not indicate the influence of sex on PA parameters. Step count and MVPA were previously found to be significantly lower in women with active RA than in HC [12]. Results of our study confirmed this finding and extended it to a male population, too, given the absence of sex differences.

### Strengths and limitations

Strengths of the present study include objective assessment of PA using accelerometry, which is preferred over questionnaires, and measurement of PA at different days across the week and at different times across the day. Since our results point towards some exacerbated differences in PA levels between pwRA and HC on week evenings and weekend days during daytime, we recommend that future research investigating accelerometry-derived PA in this population collects data on at least 1 weekday and 1 weekend day. Another strength is that we included an age-, sex-, and BMI-matched control group, providing insights into the way in which PA patterns of pwRA differ from the general population.

A limitation of this study is the interindividual variability in accelerometer wear time. Although only days with at least 10 hours of wear time were included, some participants wore the device for over 15 hours while others just met the minimum requirement. As a result, PA data may be incomplete for those with shorter wear time, potentially misrepresenting their activity levels. Additionally, data recorded between midnight and 6:30 AM were excluded to remove falsely registered wear during sleep. However, this may have led to the loss of valid data from early risers or those active after midnight. Consequently, PA and wear time could be underestimated for participants who followed the instructions but were active outside the filtered timeframe. The midnight to 6:30 AM filter was applied based on visual inspection, which revealed that some participants wore the device overnight, contrary to instructions. Including all overnight data would have introduced more bias than excluding some valid activity. Importantly, this filtering approach allowed the inclusion of all participants in the analysis, ensuring consistent data handling without the need for complete exclusions.

A minor limitation regarding the use of the MFIS questionnaire should be noted. The MFIS was originally intended to be used in patients with multiple sclerosis (MS), and although the validity and reliability of the questionnaire have been shown for patients with MS [33], this has not been addressed for pwRA. However, the current study did not focus on diagnostics and used the instrument solely to gain insight into subjectively reported fatigue in relation to objectively measured PA. The influence of limitations related to this questionnaire on the currently reported results is therefore expected to be minimal. Another limitation arises from the absence of data on participants’ employment status. While it was part of the study protocol to collect this data, only 23 out of the 41 participants answered this question. Of the 22 pwRA who answered the question, 12 were employed. The only HC who answered the question was also employed. Given the absence of data on employment status in 17 out of 18 HC individuals, it was not possible to add the employment status variable to the GEE models for an investigation of the effect of employment status on PA outcomes, or to study any differences in this effect between pwRA and HC through an interaction. Finally, the population included in the present study was relatively small, and the absence of statistically meaningful interactions between group (pwRA vs HC) and fatigue may therefore have resulted from a limitation in statistical power, rather than the true absence of interactions.

### Clinical implications

Although our results consistently show lower PA levels in pwRA than in HC, this does not necessarily imply that pwRA do not meet the World Health Organization (WHO) guidelines for PA (ie, 150-300 minutes of moderate-intensity PA or 75-150 minutes of vigorous-intensity PA throughout the week, with additional muscle-strengthening activities at minimally moderate intensity) [8]. In this study, pwRA performed 25 ± 30 minutes MVPA on weekdays, and 20 ± 30 minutes MVPA on weekend days, indicating that some pwRA do meet the WHO criteria. PA differences between pwRA and HC were largest on weekday evenings and weekend days, but further research is required to determine whether this finding was due to the need to rest because of excessive fatigue. At present, it remains unclear to what extent the observed differences in PA between pwRA and HC are clinically relevant, and further research is also needed to establish thresholds of meaning, for example, by determining the clinically meaningful levels of PA and whether they differ between RA and healthy persons. BMI management appears relevant, given the negative associations that were found between BMI and PA outcomes. Patients’ self-reported levels of fatigue should, however, be investigated longitudinally, as the results of the present study do not inform us of potential causal relationships between fatigue and PA, and therefore do not indicate whether fatigue limits PA or whether PA induces fatigue.

Using accelerometry, we found lower levels of the PA parameters VM_CPM_, step count, and MVPA in pwRA than in HC, regardless of the day of the week or the time of the day. We did not find significant group-by-time interactions, indicating a similar negative effect of having RA on PA levels at any day or time, though we cannot exclude that we lacked power for such interaction analyses. Fatigue and BMI were negatively associated with PA, but as there were no interactions between RA status and fatigue or BMI, this effect was not exacerbated in pwRA. Further research is required to distinguish the effects of transient fatigue vs fatigue as a trait, employment status, inflammation, and medication usage on PA. This will help to understand which patients with RA are most prone to fall short of the PA recommendations and to design effective interventions accordingly.

## Editor disclosure

The peer review process did not involve Editorial Board Member Sofia Ramiro, and the editorial decision-making was led by editors who were not involved in the creation of this manuscript.

## CRediT authorship contribution statement

**Maartje Cox:** Writing – review & editing, Writing – original draft, Visualization, Formal analysis, Data curation. **Sofia Ramiro:** Writing – review & editing, Supervision, Investigation, Conceptualization. **Kyra Theunissen:** Writing – review & editing, Methodology, Investigation, Conceptualization. **Kenneth Meijer:** Writing – review & editing, Supervision, Methodology, Conceptualization. **Annick Timmermans:** Writing – review & editing, Methodology, Investigation, Conceptualization. **Annelies Boonen:** Writing – review & editing, Supervision, Methodology, Investigation, Funding acquisition, Conceptualization. **Guy Plasqui:** Writing – review & editing, Supervision, Project administration, Methodology, Conceptualization.

## Competing interests

The authors declare no conflicts of interest.

## Appendix Supplementary materials

Image, application 1

## Footnotes

Supplementary material associated with this article can be found, in the online version, at doi:10.1016/j.ero.2026.100185.
