# Supplementary material for: Similar negative effects of fatigue on physical activity in persons with rheumatoid arthritis and healthy controls: results of a cross-sectional patient-control study
Source: EULAR Rheumatol Open. 2026 May 7;2(2):100185. doi: 10.1016/j.ero.2026.100185 (PMC13425159; doi:10.1016/j.ero.2026.100185)
Supplement: Supplementary file 1 [file mmc1.docx]

SUPPLEMENTARY FILE FOR:

**Similar Negative Effects of Fatigue on Physical Activity in Persons with Rheumatoid Arthritis and Healthy Controls: Results of a Cross-Sectional Patient-Control Study**

Running head: Effects of Fatigue on Physical Activity in Persons with Rheumatoid Arthritis Compared to Healthy Controls

Maartje Cox (ORCID 0009-0003-7870-9183)^1,2^; Sofia Ramiro (ORCID 0000-0002-8899-9087)^3,4^; Kyra Theunissen^1,5,6,7^; Kenneth Meijer (ORCID 0000-0001-8236-8754)^1^; Annick Timmermans^7^; Annelies Boonen^5,6^ (ORCID 0000-0003-0682-9533); Guy Plasqui (ORCID 0000-0003-4629-6479)^1^

1. Department of Nutrition and Movement Sciences, School of Nutrition and Translational Research in Metabolism, Maastricht University, Maastricht, The Netherlands
2. Australian National Phenome Centre, Health Futures Institute, Harry Perkins Institute of Medical Research, Murdoch University, Perth, Western Australia, Australia
3. Department of Rheumatology, Leiden University Medical Center, Leiden, The Netherlands
4. Department of Rheumatology, Zuyderland Medical Centre Heerlen, Heerlen, The Netherlands
5. Department of Internal Medicine, Division of Rheumatology, Maastricht University Medical Center, Maastricht, The Netherlands
6. Department of Health Services Research, Care & Public Health Research Institute (CAPHRI), Maastricht University, Maastricht, The Netherlands
7. REVAL Rehabilitation Research Center, REVAL, Faculty of Rehabilitation Sciences, Hasselt University, Hasselt, Belgium

**Corresponding author:** Maartje Cox ([maartje.cox@murdoch.edu.au](mailto:maartje.cox@murdoch.edu.au)); Australian National Phenome Centre, Harry Perkins Institute of Medical Research, 5 Robin Warren Dr, Murdoch WA 6150

| **Table S1:** Overview of the accelerometer outcomes overall and separated per day of the week (i.e., week or weekend day) | | | | | | |
| --- | --- | --- | --- | --- | --- | --- |
|  | **Overall** | | **Week** | | **Weekend** | |
| **Group** | **pwRA** | **HC** | **pwRA** | **HC** | **pwRA** | **HC** |
| **VMcpm** (mean cpm/h) | 1233.4 (509.4) | 1701.2 (492.8) * | 1261.7 (497.7) | 1673.4 (509.5) * | 1148.4 (559.7) | 1764.2 (729.5) * |
| **Step count** (steps/h) | 586.9 (225.8) | 723.8 (224.1) * | 601.5 (234.3) | 713.8 (231.8) | 543.8 (225.0) | 746.1 (246.6) * |
| **MVPA** (min/h) | 1.6 (1.4) | 2.7 (1.5) * | 1.7 (1.4) | 2.8 (1.5) * | 1.3 (1.5) | 2.4 (1.9) * |
| Data displayed as mean (SD)  * p < 0.05 between HC and pwRA  HC, healthy control; MVPA, moderate to vigorous physical activity; pwRA, persons with rheumatoid arthritis; VMcpm, vector magnitude counts per minute. | | | | | | |

| **Table S2:** Overview of the accelerometer outcomes overall and separated per time of the day (i.e., daytime or evening) | | | | |  |
| --- | --- | --- | --- | --- | --- |
|  | **Daytime** | | **Evening** | | |
| **Group** | **pwRA** | **HC** | **pwRA** | **HC** | |
| **VMcpm** (mean cpm/h) | 1494.6 (749.2) | 2009.1 (932.3) * | 801.9 (434.0) | 1242.7 (526.4) * | |
| **Step count** (steps/h) | 700.3 (290.1) | 852.1 (322.8) * | 394.7 (227.5) | 524.2 (197.5) * | |
| **MVPA** (min/h) | 2.1 (2.1) | 3.2 (2.5) * | 0.6 (0.9) | 1.6 (1.6) * | |
| Data displayed as mean (SD)  * p < 0.05 between HC and pwRA  HC, healthy control; MVPA, moderate to vigorous physical activity; pwRA, persons with rheumatoid arthritis; VMcpm, vector magnitude counts per minute. | | | | |  |

| **Table S3:** Overview of the average accelerometer outcomes separated per day of the week (i.e., week or weekend day) and per time of the day (i.e., daytime or evening) | | | | | | | | |
| --- | --- | --- | --- | --- | --- | --- | --- | --- |
|  | **Week-Daytime** | | **Week-Evening** | | **Weekend-Daytime** | | **Weekend-Evening** | |
| **Group** | **pwRA** | **HC** | **pwRA** | **HC** | **pwRA** | **HC** | **pwRA** | **HC** |
| **VMcpm** (mean cpm/h) | 1534.3 (691.3) | 1802.6 (610.6) | 880.7 (491.4) | 1457.3 (572.7) * | 1453.1 (819.7) | 2227.7 (1162.9) * | 719.5 (357.3) | 1015.6 (365.6) * |
| **Step count** (steps/h) | 727.0 (289.0) | 785.0 (292.2) | 427.5 (253.9) | 594.0 (197.6) * | 672.5 (295.4) | 923.2 (346.7) * | 360.4 (196.2) | 450.3 (173.4) |
| **MVPA** (min/h) | 2.3 (2.0) | 3.1 (1.8) | 0.9 (1.1) | 2.4 (1.6) * | 2.0 (2.3) | 3.4 (3.1) | 0.4 (0.6) | 0.7 (1.1) |
| Data displayed as mean (SD)  * p < 0.05 between HC and pwRA  HC, healthy control; MVPA, moderate to vigorous physical activity; pwRA, persons with rheumatoid arthritis; VMcpm, vector magnitude counts per minute. | | | | | | | | |

| **Table S4:** Effect of RA vs HC on step count in univariable and multivariable Generalized Estimating Equations analyses. | | | |
| --- | --- | --- | --- |
|  | **Univariable model** | **Multivariable model with FSS** | **Multivariable model with MFIS physical** |
| **Variables** | **Coefficient (95% CI)** | **Coefficient (95% CI)** | **Coefficient (95% CI)** |
| **Age** (years) | 2.48 (-3.20; 8.17) | na | na |
| **Male vs Female** | -71.13 (-206.80; 64.55) | na | na |
| **BMI** (kg/m^2^) | -20.68 (-34.90; -6.47) | -13.98 (-22.91; -5.06) | -15.29 (-24.68; -5.91) |
| **RA vs HC** | -136.89 (-272.04; -1.74) | -0.46 (-156.68; 155.76) | -33.58 (-186.91; 119.76) |
| **FSS (0-7)** | -50.15 (-92.23; -8.07) | -43.59 (-83.14; -4.05) | - |
| **MFIS physical (0-36)** | -7.22 (-13.05; -1.39) | - | -5.39 (-11.57; 0.79) |
| **MFIS cognitive (0-40)** | -5.23 (-11.77; 1.31) | na | na |
| **Day (Weekend vs Week)** | -21.39 (-130.01; 87.23) | § | § |
| **Timing (Evening vs Daytime)** | -315.38 (-398.94; -231.83) | § | § |
| **Day-Timing:** |  |  |  |
| **Weekend-Daytime** | Reference | Reference | Reference |
| **Weekday-Daytime** | -17.64 (-155.92; 120.64) | -6.03 (-147.16; 135.10) | -6.03 (-147.31; 135.26) |
| **Weekday-Evening** | -297.14 (-425.40; -168.88) | -302.06 (-425.54; -178.58) | -302.06 (-427.02; -177.09) |
| **Weekend-Evening** | -382.19 (-502.44; -261.95) | -381.98 (-500.61; -263.35) | -381.98 (-501.70; -262.26) |
| § Variables Day (Weekend vs Week) and Timing (Daytime vs Evening) were not considered for the multivariable model, since the variable Day-Timing combines the information from both these variables.  na: variable not significant in the univariable model and not a confounder for RA vs HC and therefore not included in the multivariable model  BMI, Body Mass Index; FSS, Fatigue Severity Scale; MFIS, Modified Fatigue Impact Scale. | | | |

| **Table S5:** Effect of RA vs HC on the amount of time that participants engaged in physical activity at moderate-to-vigorous intensity (MVPA) in univariable and multivariable Generalized Estimating Equations analyses stratified by age category | | | | | |
| --- | --- | --- | --- | --- | --- |
|  | **Univariable model** | **Multivariable model with FSS**  Younger participants | **Multivariable model with FSS**  Older participants | **Multivariable model with MFIS physical**  Younger participants | **Multivariable model with MFIS physical**  Older participants |
| **Variables** | **Coefficients (95% CI)** | **Coefficients (95% CI)** | **Coefficients (95% CI)** | **Coefficients (95% CI)** | **Coefficients (95% CI)** |
| **Age** (years) | 0.02 (-0.01; 0.05) | - | - | - | - |
| **Male vs Female** | 0.57 (-0.60; 1.73) | na | na | na | na |
| **BMI** (kg/m^2^) | -0.11 (-0.19; -0.03) | 0.06 (-0.18; 0.29) | -0.09 (-0.14; -0.04) | 0.06 (-0.18; 0.29) | -0.10 (-0.15; -0.05) |
| **RA vs HC** | -1.11 (-1.97; -0.25) | -1.89 (-3.26; -0.51) | -0.25 (-1.24; 0.74) | -1.67 (-2.97; -0.38) | -0.56 (-1.64; 0.52) |
| **FSS** | -0.26 (-0.52; -0.01) | 0.23 (-0.16; 0.61) | -0.20 (-0.40; -0.01) | - | - |
| **MFIS physical** | -0.04 (-0.08; 0.01) | - | - | 0.02 (-0.03; 0.07) | -0.02 (-0.07; 0.03) |
| **MFIS cognitive** | -0.04 (-0.08; 0.00) | na | na | na | na |
| **Day (Weekend vs Week)** | -0.38 (-1.11; 0.34) | § | § | § | § |
| **Timing (Evening vs Daytime)** | -1.59 (-2.18; -1.00) | § | § | § | § |
| **Day-Timing:** |  |  |  |  |  |
| **Weekend-Daytime** | Reference | Reference | Reference | Reference | Reference |
| **Weekday-Daytime** | 0.06 (-0.98; 1.11) | -0.10 (-1.78; 1.59) | 0.25 (-0.91; 1.42) | -0.10 (-1.79; 1.60) | 0.25 (-0.93; 1.43) |
| **Weekday-Evening** | -1.20 (-2.16; -0.24) | -1.42 (-2.96; 0.13) | -1.19 (-2.19; -0.19) | -1.42 (-2.96; 0.13) | -1.19 (-2.20; -0.18) |
| **Weekend-Evening** | -2.08 (-2.97; -1.19) | -2.30 (-3.78; -0.82) | -1.92 (-2.88; -0.95) | -2.30 (-3.78; -0.82) | -1.92 (-2.88; -0.95) |
| § Variables Day (Weekend vs Week) and Timing (Daytime vs Evening) were not considered for the multivariable model, since the variable Day-Timing combines the information from both these variables.  na: variable not significant in the univariable model and not a confounder for RA vs HC and therefore not included in the multivariable model  BMI, Body Mass Index; FSS, Fatigue Severity Scale; MFIS, Modified Fatigue Impact Scale. | | | | | |
